# Killer B Lymphocytes and Their Fas Ligand Positive Exosomes as Inducers of Immune Tolerance

**DOI:** 10.3389/fimmu.2015.00122

**Published:** 2015-03-20

**Authors:** Steven K. Lundy, Matthew W. Klinker, David A. Fox

**Affiliations:** ^1^Department of Internal Medicine-Rheumatology, University of Michigan Medical School, Ann Arbor, MI, USA; ^2^Graduate Training Program in Immunology, University of Michigan Medical School, Ann Arbor, MI, USA

**Keywords:** immune regulation, autoimmunity, allergy, transplantation, apoptosis, T_H_ cells, EBV

## Abstract

Induction of immune tolerance is a key process by which the immune system is educated to modulate reactions against benign stimuli such as self-antigens and commensal microbes. Understanding and harnessing the natural mechanisms of immune tolerance may become an increasingly useful strategy for treating many types of allergic and autoimmune diseases, as well as for improving the acceptance of solid organ transplants. Our laboratory and others have been interested in the natural ability of some B lymphocytes to express the death-inducing molecule Fas ligand (FasL), and their ability to kill T helper (T_H_) lymphocytes. We have recently shown that experimental transformation of human B cells by a non-replicative variant of Epstein-Barr virus (EBV) consistently resulted in high expression of functional FasL protein. The production and release of FasL^+^ exosomes that co-expressed major histocompatibility complex (MHC) class II molecules and had the capacity to kill antigen-specific T_H_ cells was also observed. Several lines of evidence indicate that FasL+ B cells and FasL^+^MHCII^+^ exosomes have important roles in natural immune tolerance and have a great deal of therapeutic potential. Taken together, these findings suggest that EBV-immortalized human B lymphoblastoid cell lines could be used as cellular factories for FasL^+^ exosomes, which would be employed to therapeutically establish and/or regain immune tolerance toward specific antigens. The goals of this review are to summarize current knowledge of the roles of FasL^+^ B cells and exosomes in immune regulation, and to suggest methods of manipulating killer B cells and FasL^+^ exosomes for clinical purposes.

## Introduction

Fas ligand (FasL, CD178) is an important mediator of peripheral immune tolerance that is constitutively expressed by specialized epithelial cells at immune privileged sites of the body such as the eye and reproductive organs (1–3). Within the immune system, FasL expression is most often attributed to cytotoxic CD8^+^ T lymphocytes (CTL) and natural killer (NK) cells that are activated in response to viral infections or malignancies (4–8). A requirement for FasL expression by CD8^+^ veto T cells leading to deletion of effector CTL has been demonstrated to enhance tolerance of transplanted bone marrow cells (4, 9). Interaction of FasL with its receptor Fas (CD95) initiates a signaling cascade that leads to programmed cell death (apoptosis) in susceptible target cells (10, 11). The importance of FasL–Fas-mediated apoptosis to immune regulation was first highlighted by studies of mice with mutations affecting this pathway that developed lymphoproliferative disorders and produced autoantibodies similar to those found in systemic lupus erythematosus (SLE) patients (12, 13). In humans, defects in the FasL–Fas signaling pathway result in the autoimmune lymphoproliferative syndrome (ALPS), which most often manifests as autoimmune hemolytic anemia or other cytopenias caused by cell-specific autoantibody production (14). Thus, it is clear that FasL–Fas interactions play a significant role in regulating the production of pathogenic autoantibodies. It is well-established that mature B lymphocytes that have been activated through interactions with T helper (T_H_) lymphocytes are susceptible to FasL-mediated apoptosis, and presumably receive this signal from the interacting T_H_ cell (15–17). What has often been overlooked is that some B lymphocytes express functional FasL constitutively, and that many types of stimuli can induce FasL expression in B cells (18). Therefore, it is important to consider that the reciprocal induction of apoptosis mediated by the B cell against the T_H_ cell during these cognate interactions may also play a critical role in immune tolerance toward self-antigens.

KEY CONCEPT 1. Some B lymphocytes express functional FasL constitutively In mice, these have been identified primarily within the CD5^+^ B-cell subset in spleen and lung. It remains to be determined how important they are in networks of mucosal and systemic immune tolerance, and which B-cell subset(s) in humans, if any, have similar functions.

## FasL^+^ Killer B Cells

Expression of FasL by B cells was first reported in 1996 by Hahne and Tschopp, who demonstrated that activation of mouse B cells by mitogens led to FasL expression and killing of Fas-sensitive target cells by B cells from wild-type mice but not from FasL-mutant mice (19). Several other reports confirmed expression of FasL by both human and mouse B cells (20–30). Notably, it was found that human B-cell expression of FasL was induced during human immunodeficiency virus (HIV) and Epstein-Barr virus (EBV) infections, and that T_H_ cells from infected individuals were very sensitive to Fas-mediated apoptosis (21, 27). While these data suggested that FasL expression by B cells might play a pathogenic role by inducing T_H_ cell death in these viral infections, this was not explicitly tested. The first direct evidence that human B cells could induce T_H_ cell apoptosis was demonstrated using FasL^+^ B-chronic lymphocytic leukemia (B-CLL) cells to kill a susceptible CD4^+^ T-cell leukemia line (23). FasL expression is common in the aggressive form of B-CLL, and has been reported in other human B-cell leukemias and lymphomas, most notably, multiple myeloma (31–34). Important questions that remain regarding human FasL^+^ B cells include: 1) are there specific human B-cell subsets that preferentially express FasL; 2) how is FasL expression regulated in human B cells; and 3) what are the normal functions of FasL^+^ B cells in humans? Studies performed in mice may provide some answers to these questions.

Analysis of T_H_ cell apoptosis and the cellular distribution of FasL expression in the schistosome worm infection model led to our independent discovery of mouse FasL^+^ B cells (35). In that study, antigen-specific T_H_ cell apoptosis was impaired when B cells but not CD8^+^ T cells were depleted from cell cultures, and was regained when T_H_ cells were mixed with purified B cells from worm-infected mice. In a follow-up study, it was discovered that FasL expression was present on 1–2% of splenic B lymphocytes in uninfected mice, and was almost exclusively found within the CD5^+^ B-cell subset (36). The number of CD5^+^ and CD5^neg^ B cells that expressed FasL increased significantly during the acute phase of schistosome infection, paralleling increases in T_H_ cell apoptosis. Purified CD5^+^ B cells from both naïve and schistosome-infected mice had potent *in vitro* cytotoxic activity against T_H_ cells isolated from schistosome-infected mice, but not naïve T_H_ cells. In summary, mouse CD5^+^ B cells are constitutive and inducible expressers of functional FasL, and are efficient killer cells toward antigen-specific T_H_ cells *in vitro*. It is yet to be determined if the human equivalents of CD5^+^ B cells, or other human B-cell subsets with reported regulatory functions, have the same constitutive expression of FasL and ability to kill antigen-specific T_H_ cells *in vivo* (37).

## Control of Killer B Lymphocyte Growth and Function

The schistosome model is an excellent system for studying the progression of the immune response. The initial reaction to worm egg deposition is an innate, pro-inflammatory reaction followed by acute T_H_1- and T_H_17-mediated inflammation that transitions to a strong T_H_2-mediated immune response, and which ultimately culminates in a chronic, fibrotic, and systemically immunosuppressive reaction (38). Peak FasL^+^ B-cell expansion and activation in the schistosome model occurred in the latter stages of the T_H_2 response and beginning of the chronic phase (35). B cells isolated from infected mice could be further induced to express surface FasL by treatment with interleukin 4 (IL-4) and IL-10 (36). More recently, we have shown that *in vitro* activation of purified splenic B cells from naïve mice with CD40 ligand (CD40L) and the T_H_2 cytokine IL-5 (but not IL-4) was sufficient to induce the proliferation of CD5^+^FasL^+^ B cells, and to increase the peptide-specific cytotoxic activity of B cells against T_H_ cells (39). Naïve splenic B cells stimulated with the combination of CD40L and IL-5 and IL-4 were actually inhibited in their surface FasL expression and killer function, despite expressing similar levels of intracellular FasL. These data demonstrate novel differences in killer B-cell responses to the T_H_2 cytokine milieu and suggest that intracellular sequestration of FasL is a mechanism that regulates killer B-cell function.

KEY CONCEPT 2. Activation of purified splenic B cells from naïve mice with CD40 ligand (CD40L) and the T_H_2 cytokine IL-5 (but not IL-4) was sufficient to induce the proliferation of CD5^+^FasL^+^ B cells This technique will be helpful for overcoming limitations in killer B-cell numbers and for studying the signaling requirements and *in vivo* effector functions of killer B cells in the future.

**Figure 1:**
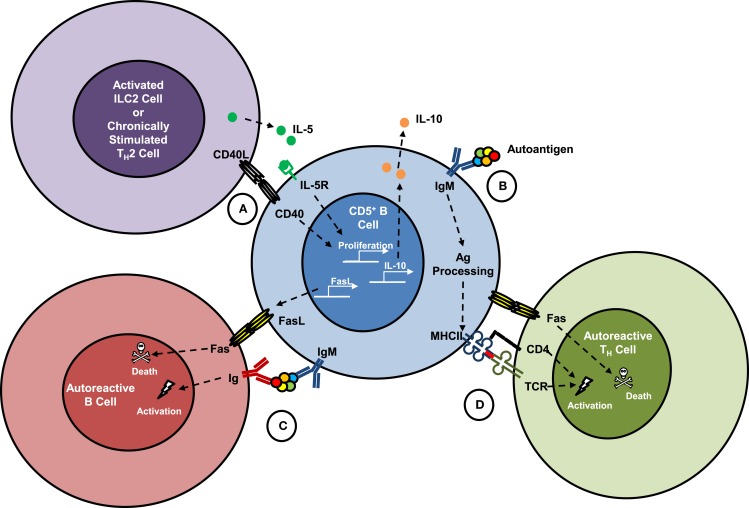
**Hypothesized interactions of killer B cells with other lymphocytes**. Fas ligand (FasL) expression is constitutive on mouse spleen and lung CD5^+^IgM^high^ B cells, which have been shown to kill antigen-specific T_H_ cells *in vitro*. Co-activation of mouse B cells through CD40 and the IL-5 receptor led to proliferation of CD5^+^ B cells, increased surface expression of FasL and killer function, and production and release of IL-10. **(A)** Interactions of killer B cells with mucosal type 2 innate lymphoid cells (ILC2), or with chronically stimulated T_H_2 cells *in vivo* would be expected to support their growth and functions, but has not been formally proven. **(B)** Surface immunoglobulins on CD5^+^ B cells are poly-reactive and are known to recognize autoantigens that once bound can be internalized and processed into peptides, which are then presented to T_H_ cells on class II major histocompatibility (MHCII) molecules. **(C)** Binding of an autoantigen simultaneously by a killer B cell and an effector B cell could be a mechanism to explain B-cell fratricide, which has been described in several reports. **(D)** Killer B-cell uptake and presentation of autoantigens to T_H_ cells in the context of FasL–Fas signaling *in vivo* could lead to activation-induced cell death and is hypothesized to be an important mechanism for maintaining peripheral tolerance and preventing autoimmune diseases. These processes may also play a role in tolerance to food antigens, other allergens, and commensal microbes.

Until recently, IL-4 and IL-5 were generally accepted as cytokines produced by T_H_2 cells that have distinct but cooperative effects in driving T_H_2-mediated inflammation. However, a report by Islam et al. showed that IL-4 is an early activation product of T_H_2 cells and that chronically activated T_H_2 cells may switch to predominant production of IL-5 (40). It has also been reported that mucosal type 2 innate lymphoid cells (ILC2 cells) produce high levels of IL-5 compared to IL-4 when stimulated by IL-25 or IL-33, and are important contributors to T_H_2 inflammation. Interestingly, CD5^+^ B cells are more abundant in the mucosa, where they are commonly referred to as B-1a cells, and are sparse in the lymph nodes or circulation. It is quite likely that B-1a cells receive signals from ILC2 cells under homeostatic and inflammatory conditions. Although it remains to be formally proven, such an interaction would be expected to support mucosa-associated FasL^+^CD5^+^ B cells (Figure 1A). This may have important implications for protection from food allergies and local mucosal inflammation, and could play a role in the broader systemic immune tolerance mediated through the mucosal immune system.

Mice with an X-linked functional mutation of Bruton’s tyrosine kinase (Xid mice) or IL-5 deficiency have a severe impairment of mucosal B-1a cells (41–43). These mice have provided useful models to study the functions of CD5^+^ B cells *in vivo*. B-1a cells are a major source of natural antibodies, which are germline IgM immunoglobulins that are present at birth and which tend to have poly-reactive specificities for carbohydrates, lipids, and nucleic acids rather than protein components of antigens (44, 45). Interestingly, many of these germline-configured IgM antibodies also have a strong tendency to bind self-antigens (Figure 1B), and for this reason, B-1a cells have often been implicated as a source of autoantibodies and as contributors to autoimmune disease pathogenesis (46). The likelihood that self-reactive CD5^+^ B cells may also constitutively express FasL suggests that their more common role in autoimmunity may be to eliminate other self-reactive B and T cells (Figures 1C,D), and thus protect against the development of autoimmune diseases (47).

## FasL^+^ B Cells and Tolerance

Immune tolerance is an active and complex process by which potentially harmful self-reactive lymphocytes are either eliminated, shunted into permanently inactive (anergic) states, or induced to become specialized immune regulatory cells. Breakdowns in tolerance that occur either centrally during lymphocyte development, or peripherally during inflammatory responses can lead to hyperactive immune reactions and severe pathological consequences (48). As evidenced by the autoimmune pathology associated with Fas or FasL-deficiencies, this death pathway is critical to normal immune tolerance (8). It is important to note that tolerance can also develop toward foreign antigens and is highly dependent on the location of antigen exposure and concurrent signals from the local environment. The fetal–maternal interface, gut mucosa, other sites of immune privilege, and the microenvironments created by tumors are especially well-suited to induction of tolerance (49). While the relative importance of FasL^+^ B cells in immune tolerance has not been studied extensively, several reports support an *in vivo* role for killer B cells in immune regulation and the induction of tolerance.

Minagawa et al. demonstrated that tolerance toward male H-Y antigen could be generated in mice through adoptive transfer of male splenic B cells into female recipients (50). The authors went on to show that tolerance was dependent on functional Fas receptor in the recipient mice, and that FasL on donor B cells was required (50). In separate studies, we showed that Xid mice, which lacked lung FasL^+^CD5^+^ B cells, failed to induce lung T_H_ cell apoptosis in a chronic airway allergen exposure model (51). Low T_H_ cell death in comparison to wild-type mice correlated with increased cytokine production, eosinophilia, and mucus production in the lungs despite high levels of IL-10 in the lung homogenates of the Xid mice (51). In the collagen-induced arthritis model, increased severity of joint inflammation correlated with decreased levels of FasL^+^ B cells in the spleen, and decreased killer function of B cells against antigen-specific T_H_ cells (47). Montandon et al. demonstrated that activation of bone marrow-derived pro-B cells via toll-like receptor 9, induced pro-B-cell FasL expression, and that adoptive transfer of these cells into non-obese diabetic (NOD) mice resulted in protection from the spontaneous development of type 1 diabetes (52). An earlier study in NOD mice had also demonstrated a protective effect of B cells that were activated by bacterial lipopolysaccharide exposure, and expressed both FasL and the anti-inflammatory cytokine, transforming growth factor beta (TGFβ) (53). Many other studies have demonstrated the ability of transferred CD5^+^ B cells to suppress immune responses and induce therapeutic tolerance, however, the focus of those reports was on the ability of CD5^+^ B cells to produce IL-10, and unfortunately, FasL production and function were not addressed (54–59). It would be interesting to repeat some of the above studies using IL-10-deficient, TGFβ-deficient, and FasL-deficient B cells to measure the relative importance of each molecule to the therapeutic efficacy of B cells.

KEY CONCEPT 3. Tolerance toward male H-Y antigen could be generated in mice through adoptive transfer of male splenic B cells into female recipients This study by Minagawa et al. demonstrated that the tolerizing effect of transferred B cells was Fas ligand-dependent. This report supports the concept that killer B cells could be targeted therapeutically to induce immune tolerance.

We have recently reported that B cells isolated from tumor-draining lymph nodes (TDLN) in a mouse breast tumor model were able to kill tumor cells *in vitro* and prevent metastases *in vivo*, and that B cells from IL-10-deficient mice were more potent killers of the tumor cells (60). FasL expression on TDLN B cells was not higher in the IL-10 knockout mice nor was there a difference in the total number of killer B cells. These data suggest that the lack of IL-10 from the adoptively transferred TDLN B cells may have had other inhibitory effects on B-cell killer functions, such as blocking critical interactions with the tumor cells and/or protective effects of IL-10 on the targeted tumor cells (60).

## Restrictions on FasL Expression

Detection of FasL expression on the cell surface is usually not very robust, and most likely only represents a fraction of the total FasL protein produced by the cell. Cell types that do have measurable FasL on their surface, including B-1a cells, are often restricted in their distribution to specific niches where their interactions with Fas-sensitive target cells are more limited than if the FasL^+^ cells were allowed to circulate systemically. In cytotoxic T cells and NK cells, surface expression of FasL is transient due to the activity of metalloproteinases that cleave FasL and release the molecule in an inactive soluble form (61). Experimental systems in which FasL expression was forced to occur on cell surfaces, or in which cleavage of cell surface FasL was inhibited, most often resulted in the induction of target cell activation leading to inflammation rather than cell death (62–67). Moreover, the levels of transcription of the FasL gene in CTL and NK cells are very low in comparison to the amount of FasL protein found within the cells. This suggests that the FasL protein is fairly stable and is stored in the cell cytoplasm in a form that could be quickly transported to the cell surface when an appropriate target cell is identified.

In 1996, Martinez-Lorenzo and colleagues demonstrated that a preformed pool of FasL protein was sequestered within the cytoplasm of the immortalized human T-cell line, Jurkat (68). Upon stimulation, the FasL was rapidly released into the culture supernatant in a form that could induce cell death of other Jurkat T cells (68). In a subsequent study, these authors showed that bioactive FasL released by Jurkat was associated with microvesicles that could be removed by ultracentrifugation, and that similar FasL^+^ microvesicles were produced by non-transformed human T cells (69). It is now understood that these microvesicles were FasL^+^ exosomes and that this finding could explain conflicting data regarding soluble FasL and its role in mediating activation-induced cell death.

KEY CONCEPT 4. Bioactive FasL released by Jurkat was associated with microvesicles that could be removed by ultracentrifugation This study and others support the concept that killer cells package much of their Fas ligand into exosomes, which limits the expression of the protein on the cell surface, yet provides a ready pool of FasL that can be quickly released upon activation.

Exosomes are extracellular vesicles that are approximately 30–100 nm in diameter, which are secreted by many types of cells, including B cells and other hematopoietic cells (70, 71). Exosomes can be found in many types of bodily fluids (blood, breast milk, urine), and ultracentrifugation is required to separate them from soluble proteins. Truly soluble FasL protein, removed from the cell surface by metalloproteinase-mediated cleavage, must now be considered separately from exosomal FasL that is membrane bound (66, 72–74). Many studies showing that soluble FasL was inhibitory of cell death were most likely influenced by the presence in the tested samples of cleaved FasL protein. Soluble FasL does not cause Fas aggregation and may block binding of membrane-associated FasL with death-inducing capacity. In contrast, exosomal FasL is very efficient at causing Fas aggregation, and may have been responsible for the results of those studies showing that “soluble” FasL was able to induce cell death. FasL^+^ exosomes are released by many types of FasL-producing cells, including NK cells, cytotoxic T cells, retinal pigment epithelial cells, and placental trophoblasts (75–77). FasL^+^ tumor cells can also produce FasL^+^ exosomes that target tumor-specific CD8^+^ T cells and aid in tumor immune escape (78). A direct demonstration that killer B cells can naturally produce FasL^+^ exosomes has not yet been reported, but it also has not been ruled out.

**Figure 2:**
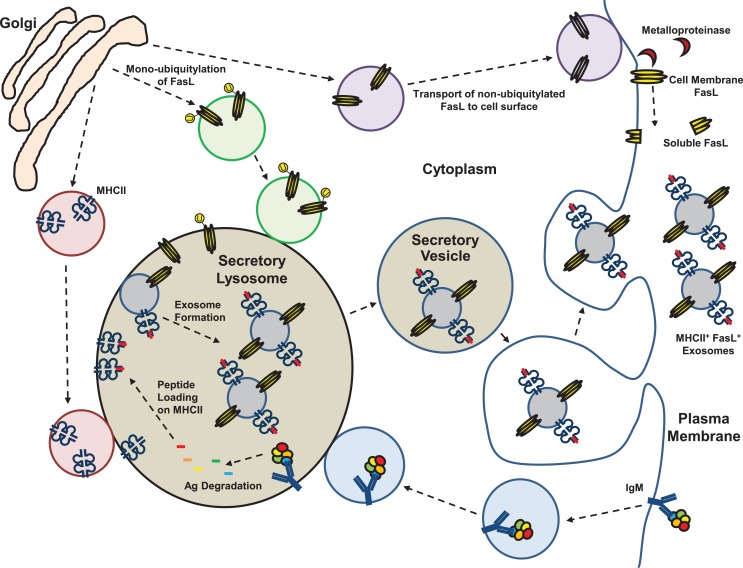
**Membrane, exosome, and soluble forms of Fas ligand**. Fas ligand (FasL) protein has been detected in several different forms in mouse and human cells and bodily fluids. Expression of FasL mRNA is generally low and the protein is assembled as a homotrimer in the trans-Golgi prior to transport to the cell surface. Plasma membrane expression of FasL is constitutive on a few specialized epithelial cell types at sites of immune privilege and on CD5^+^ B cells that have restricted localization. Cell surface expression of FasL is regulated by proteolytic cleavage by metalloproteinases, or by shunting of FasL protein into the secretory lysosomal compartment (SLC). Trafficking of FasL to the SLC is controlled by mono-ubiquitylation. The SLC is a site of antigen processing for molecules transported there from the cell surface by immunoglobulins, and processed peptides are loaded onto MHC class II (MHCII) molecules in the SLC. Through internal budding of the SLC membrane, FasL and MHCII are incorporated into exosomes that are stored in secretory vesicles of killer lymphocytes. Activation of the cell leads to their transport to the cell surface and release into the extracellular space.

FasL^+^ exosomes circulating in normal human and mouse blood can co-express class II major histocompatibility complex (MHCII) molecules that are characteristic of professional antigen-presenting cells (APC) (79, 80). In APC, exosomes originate from the secretory lysosome where newly synthesized and recycled MHCII molecules are loaded with peptides derived from endocytosed proteins (Figure 2) (81). Thus, exosomes derived from APC contain peptide-loaded MHCII and co-stimulatory molecules, and are capable of activating antigen-specific T_H_ cells (81). Unique to B cells is the fact that their APC function is restricted to antigens recognized by their surface immunoglobulins, which when bound to antigen are able to transfer the antigens directly to the secretory lysosomal compartment where MHCII^+^ exosomes are assembled (Figure 2) (82). In the case of an autoreactive B-1a cell that expresses FasL, we hypothesize that self-antigen uptake results in incorporation into MHCII^+^FasL^+^ exosomes (Figure 2). Subsequent binding of an autoreactive T_H_ cell to these autoantigen/MHCII complexes and co-ligation by FasL either during cell–cell or cell–exosome interaction could then result in T_H_ cell death and induction of immune tolerance toward that self-antigen. Similar processes could be envisioned if the B-1a cells were specific for food allergens, commensal bacteria, or other antigens toward which immune tolerance was desired. The unique positioning of B-1a cells at mucosal surfaces favors their involvement in maintaining local mucosal tolerance through surface expression of FasL and direct interaction with local cells. Beyond that, if the mucosal B-1a cells also produce FasL^+^MHCII^+^ exosomes, this could be an important yet overlooked natural mechanism by which systemic tolerance to mucosa-associated antigens is induced and maintained without the need for killer B-cell circulation outside the mucosa.

KEY CONCEPT 5. FasL^+^ exosomes circulating in normal human and mouse blood can co-express class II major histocompatibility complex (MHCII) molecules that are characteristic of professional antigen-presenting cells (APC) The origin of these naturally occurring FasL^+^MHCII^+^ exosomes has not been positively identified and we hypothesize that killer B cells are one likely source.

## FasL^+^ Exosomes and Immune Tolerance

The therapeutic application of exosomes has received increasing attention as a means to deliver signals to specific cell types, and as an alternative to adoptive immune cell therapy (83). Most of the efforts have been focused on using APC-derived exosomes to deliver activation signals for generating anti-tumor or anti-microbial immune responses (84–86). However, some investigators have focused instead on the immune-tolerizing capacity of exosomes, with particular interest placed on FasL^+^MHCII^+^ exosomes (70, 87). An early study showed that bone marrow-derived dendritic cells (BMDCs) genetically engineered to express FasL were able to produce FasL^+^MHCII^+^ exosomes that had direct killing effects on CD4^+^ T_H_ cells (88). Further studies revealed the presence of naturally occurring FasL^+^MHCII^+^ exosomes in the blood of immunized mice, confirming the production of FasL^+^MHCII^+^ exosomes by non-transduced cells (80). Endogenously -produced FasL^+^MHCII^+^ exosomes mediated antigen-specific suppression of delayed-type hypersensitivity and experimental arthritis upon transfer to recipient mice (80). These naturally occurring FasL^+^MHCII^+^ exosomes did not express markers typical of BMDC-derived exosomes but did express CD11b, a surface molecule expressed by monocytes, macrophages, and mucosal B-1 cells. FasL^+^MHCII^+^ exosomes have also been found in human plasma, and had FasL-dependent suppressive activity against CD4^+^ T cells (79). To date, human FasL^+^ killer B cells have not been positively identified, making it difficult to define markers that would allow one to conclude whether or not they are the source of naturally -occurring human FasL^+^MHCII^+^ exosomes. However, one of our recent investigations demonstrated a very robust method for experimentally producing human B- cell-derived FasL^+^MHCII^+^ exosomes.

## Human B Cells Transformed by EBV Produce FasL^+^ Exosomes

**Figure 3:**
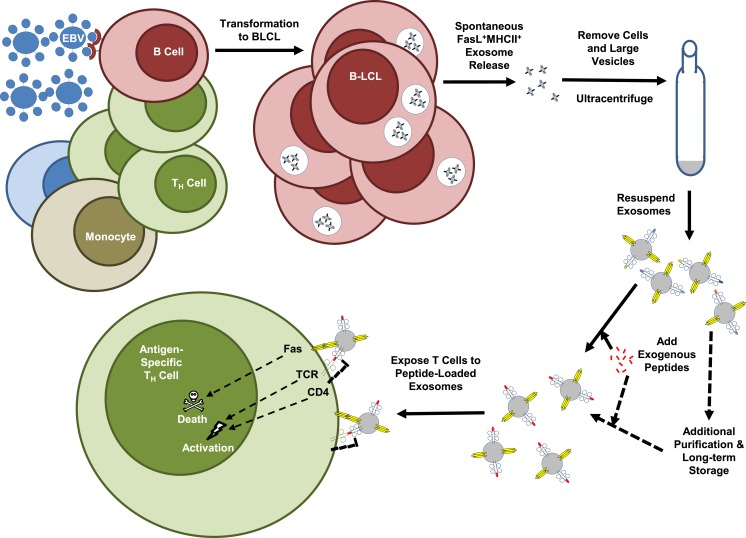
**FasL^+^MHCII^+^ exosome production by EBV-transformed B-LCL**. Epstein-Barr virus (EBV) has been used for many years to experimentally transform human B cells into immortalized B lymphoblastoid cell lines (B-LCL). The figure is a schematic of the methods recently employed to demonstrate the killer function of B-LCL-derived exosomes. Use of the non-replicative laboratory EBV strain, B95-8, resulted in consistent induction of FasL expression by transformed B-LCL. These B-LCL packaged FasL along with MHC class II molecules into exosomes that were then spontaneously released into supernatants of the B-LCL cultures grown in exosome-free media. Multiple rounds of centrifugation at increasing speeds removed cells, debris, and larger vesicles from the culture supernatant, which was then spun at 110,000 × *g* to pellet the exosome fraction. Resuspended exosomes were exposed to exogenous tetanus toxoid peptide or a bacterial superantigen, washed, and then added to cultures of autologous T_H_ cells. The method resulted in T_H_ cell death that was exosome and antigen-dependent, and FasL-mediated as demonstrated by the use of FasL-neutralizing antibodies.

In attempting to identify human B cells that produce FasL, our laboratory screened a panel of 20 EBV-transformed B lymphoblastoid cell lines (B-LCL) that had been produced using the non-replicative laboratory B95-8 EBV strain (89). This virus has been used for decades to produce immortalized human B-LCL for studies of antibody rearrangement and other B-cell functions, and there are likely to be thousands of B-LCL distributed throughout the world. Although individual B-LCL had previously been demonstrated to express functional FasL, it was remarkable to find that every one of the lines we tested expressed a high amount of FasL protein. Despite easily detectable amounts of protein as measured by Western blots of whole cell lysates and intracellular staining, FasL protein was not present on the cell surface of the B-LCL (89). In keeping with what was noted above for other cell types that express FasL, we found that the B-LCL spontaneously produced and released FasL^+^MHCII^+^ exosomes that could be purified from the cell culture supernatant by ultracentrifugation (Figure 3) (89). The B-LCL exosomes were then used to induce death of autologous T_H_ cells through antigen-dependent and FasL-mediated mechanisms as demonstrated by the use of bacterial superantigen or an exogenously loaded tetanus toxoid peptide in the presence or absence of FasL-neutralizing antibodies (Figure 3) (89). We believe that these findings open up the possibility that B-LCL could be used as cellular factories for producing FasL^+^MHCII^+^ exosomes for use in patient-customized treatments for many types of hyper-inflammatory conditions.

KEY CONCEPT 6. B-LCL spontaneously produced and released FasL^+^MHCII^+^ exosomes that could be purified from the cell culture supernatant by ultracentrifugation These exosomes had potent killer function *in vitro* against human TH cells that was both antigen- and FasL-dependent. The fact that every B-LCL that was tested did this suggests that EBV could be used to create FasL^+^ exosomes from any individual’s B cells.

## Advantages of B-LCL Exosomes for Therapy

Cellular immunotherapy mediated by adoptive transfer of regulatory lymphocytes has been shown to suppress autoimmune diseases and allergic responses in many mouse models (90–92). These methods hold promise and have given us tools to dissect the importance of the different cell types and factors involved in immune regulation. However, many large hurdles remain in the translation of cellular immunotherapy into common clinical practice. Perhaps, the most important of these obstacles is that the transferred cells must be closely matched or identical to the MHC of the recipient in order to not be rejected. Ideally, this could be overcome if the transferred cells were autologous. The regulatory cells would have to be purified from patient blood, expanded in culture, quality-tested, and then reinfused in such a way that they would be viable and have the desired therapeutic effect. This form of treatment would be very expensive and time consuming. Notably, a defect in the function of regulatory T or B cells may be central to the cause of the inflammatory condition being treated, and therefore, infusion of more of these flawed cells may not be therapeutically helpful. Live regulatory cells may also turn off regulatory functions after transfer, or worse, change phenotype and participate in the inflammatory reaction. Much important work is being done and should continue in order to solve these problems and make adoptive cellular immunotherapy a better therapeutic option.

As subcellular alternatives to regulatory cell transfers, FasL^+^MHCII^+^ exosomes may hold some distinct advantages. First among these is that the killing of target T_H_ cells by exosomes is not dependent on the presence of other cells. They also can be tailored to be antigen-specific and would be expected to only target a small subset of T_H_ cells, while sparing desirable T_H_ cells and protective immunity toward pathogenic microbes. Cell death induction by its nature is also likely to have more permanent or long-lasting effects in comparison to transfer of cells that express IL-10 or TGFβ which might only transiently modify the immune response. Exosomes lack the cellular machinery to switch their functions after transfer, and therefore, are much less likely to be turned off or to switch to a pathogenic role than are transferred regulatory cells (93). Exosomes have been reported to be functional even after long-term storage in low temperature freezers. This would suggest that once a batch of FasL^+^MHCII^+^ exosomes has been produced, isolated, and quality-tested, they could be banked in repositories until needed.

The EBV-transformation system that we utilized may provide some additional improvements over other methods of producing FasL^+^MHCII^+^ exosomes. The non-replicating, laboratory strain of the virus seems to be very efficient at transforming human B cells from any donor and activating their natural ability to express FasL. In our experience, these cells will grow indefinitely in cell culture and require relatively little labor to keep them healthy and producing exosomes. B-LCL can be frozen and stored in liquid nitrogen for decades without losing the ability to grow in cell culture. Transformation of the patient’s own B cells would allow for long-term propagation of the cells, scaling up of the culture as needed, and thus a potentially unlimited supply of FasL^+^ exosomes that would be 100% MHC-matched, and therefore, tolerated upon reinfusion into the patient. The plethora of lines that have already been transformed, frozen, and stored also provides a large potential repository of B-LCL that may closely match the MHC of the recipient patients, which could circumvent unexpected obstacles that might occur with transformation of a specific individual’s B cells.

KEY CONCEPT 7. A potentially unlimited supply of FasL^+^ exosomes that would be 100% MHC-matched, and therefore, tolerated upon reinfusion into the patient Clinical translation of adoptive immunotherapy, whether it be cellular or vesicular, would be streamlined if mismatched MHC could be avoided. The exosome production method we describe may overcome this and other important obstacles.

Unlike the genetic manipulations that were required to force FasL expression in dendritic cells, the B-LCL spontaneously produce FasL^+^MHCII^+^ exosomes. By taking advantage of the natural ability of B cells to express functional FasL, EBV may promote its own survival and ability to persist in infected individuals. The fact that the non-replicating B95-8 laboratory strain is capable of driving FasL^+^MHCII^+^ exosome production is especially important since there are no active virions in the B-LCL culture, and therefore, little to no danger of transferring replicating EBV along with the exosome infusion. Although the direct application of B-LCL-generated FasL^+^ exosomes for use as immune-tolerizing agents in humans has not been tested, and is likely to be far off, the promising results from mouse models and their advantages over cellular immunotherapy as outlined above suggest that such a strategy may work and is worth further exploration.

## Engineering EBV for Exosome Therapies

**Figure 4:**
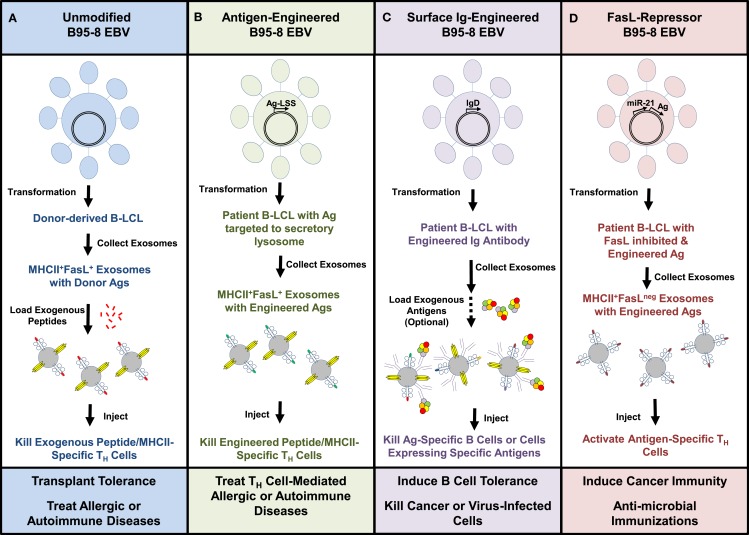
**Potential therapeutic uses of B-LCL exosomes**. Exosomes isolated from culture supernatants of EBV-transformed B-LCL were able to kill antigen-specific T_H_ cells *in vitro* through a FasL-dependent mechanism. This schematic shows ways in which the B95-8 EBV strain might be used in its native form or genetically modified to manufacture customized B-LCL exosomes. **(A)** Unmodified virus could be used to generate autologous or allogeneic B-LCL that spontaneously produce FasL^+^MHCII^+^ exosomes. Replacement of MHC-bound peptides with exogenous peptides could be used to tailor the therapy toward specific antigens. **(B)** An alternative would be to modify the EBV genome to create B-LCL that express specific antigens linked to a lysosomal sorting sequence (LSS), which would target the proteins to the secretory lysosome where exosomes are assembled. **(C)** EBV engineered to express rearranged surface immunoglobulins (IgD or IgM) with known specificities onto exosomes may prove useful in targeting antigen-specific B cells, cancer, or virus-infected cells. **(D)** Inhibition of FasL expression in B-LCL through the repressor miR-21 or other molecules introduced into the EBV genome would be expected to yield FasL^neg^ exosomes that could be used as vaccine adjuvants. Combining these genetic manipulations may further enhance the therapeutic potential of B-LCL-derived exosomes.

We envision that someday B-LCL-derived exosomes, such as those employed in our previous study, could be useful in several clinical applications. First, using the existing methods and the B95-8 strain of EBV, we would propose transforming B cells from the patients to be treated, isolating FasL^+^MHCII^+^ exosomes from cell cultures of these B-LCL and then loading them with exogenous peptides specific to the disease being targeted prior to infusion of autologous exosomes back into the patient (Figure 4A). Such a strategy, if successful, may be useful in treating allergies or autoimmune diseases in which the dominant antigens are identifiable. A similar strategy might also be used to tolerize a transplant recipient toward tissue from an allogeneic organ donor. In this approach, FasL^+^MHCII^+^ exosomes from B-LCL of a potential organ donor would be loaded with tissue peptides and infused into the recipient in order to induce death of donor MHC-specific T_H_ cells (Figure 4A).

KEY CONCEPT 8. Someday B-LCL-derived exosomes, such as those employed in our previous study, could be useful in several clinical applications The use of the non-replicative EBV strain to manufacture exosomes may mitigate some of the safety issues associated with this type of therapy, and also provide interesting opportunities to customize the exosomes through genetic engineering of the virus.

Beyond these uses of the existing EBV strain to produce therapeutic FasL^+^MHCII^+^ exosomes, we believe that there could be value in genetically modifying the B95-8 strain in order to further enhance the capabilities of B-LCL-derived exosomes. The genome of EBV is maintained in proliferating LCLs as a large episome (~167 kb), and techniques for engineering recombinant EBV are well-established (94, 95). Using a lysosomal sorting sequence fused to the protein of interest, the inserted gene products could be actively targeted into the secretory lysosomal compartment where FasL^+^MHCII^+^ exosomes are assembled and readied for transport to the cell surface (Figure 4B). Such a technique could be useful for targeting allergen proteins or autoantigens into the exosomal pathway as a potential improvement over loading cells or exosomes with exogenous peptides.

One means of improving the binding of exosomes to a desired target cell or tissue could be to engineer in a recombined immunoglobulin gene with a known specificity so that it is expressed on the exosome surface (Figure 4C). Expression of IgM and IgD has been found on the surface of naturally produced B-cell-derived exosomes (96). This technique may be especially useful for targeting other B lymphocytes with specificity for the same antigen, or for directing FasL^+^ exosomes to attack tumor cells or virus-infected cells. As described in a previous section, a recent study in mice demonstrated the ability of adoptively transferred FasL^+^ B cells to directly kill breast tumor cells and limit tumor metastases (60).

Finally, there are circumstances in which it might be desirable to be able to manufacture MHCII^+^ exosomes that do not express FasL. There is a great deal of interest in developing exosomes as vaccine adjuvants to induce T-cell responses against cancers and pathogenic microbes. We believe that the transformed B-LCL could provide a nearly unlimited source of individualized exosomes if the EBV virus could be engineered to repress instead of promote the expression of FasL (Figure 4D). One scenario would be to insert the coding sequence for micro RNA-21 (miR-21), a repressor of FasL expression, into the EBV genome (97, 98). The relative ease of creating and maintaining an immortalized B-LCL that naturally produces FasL^neg^MHCII^+^ exosomes could be a fundamental improvement over the use of non-transformed dendritic cells. Such an EBV virus could also be further manipulated genetically to express specific tumor antigens or microbial products to enhance the immunizing effects of the FasL^neg^ B-LCL exosomes (Figure 4D).

## Conclusion

There is still a great deal of basic and translational research to be done before we can attempt to manipulate killer B cells or their FasL^+^ exosomes in clinical trials. Some of the major questions that remain are: (1) is it safe to boost killer B-cell function *in vivo* and (2) what is the best approach toward achieving that goal. Fundamental to this pursuit will be better knowledge of how killer B cells are normally regulated in the body, their role in maintaining immune homeostasis and preventing disease, and the interactions they have with other regulatory or pro-inflammatory immune cells. It may be that many of the current therapies and medications used to treat autoimmune diseases or allergies have an effect on killer B cells that has gone unnoticed. In the case of development of B-LCL exosomes for therapy, it is likely that new techniques and equipment will have to be developed in order to reliably scale-up production and purify B-LCL-derived exosomes to the point that they can be tested for safety and efficacy in human subjects.

Research focused on immune regulatory pathways has yielded a wealth of knowledge and a number of potential targets for therapy. Beyond the established roles of regulatory T cells and their signature cytokines IL-10 and TGFβ in immune regulation, it has become increasingly clear that a larger network of cells and factors exists that induce and maintain immune tolerance. Increasing evidence suggests that killer B cells and FasL^+^MHCII^+^ exosomes play important and potentially irreplaceable roles in immune tolerance networks as specific regulators of T_H_ cells. By increasing awareness of killer B cells and their therapeutic potential, we hope to foster interest in studying their biology and roles in human health and disease.

## Conflict of Interest Statement

The authors declare that the research was conducted in the absence of any commercial or financial relationships that could be construed as a potential conflict of interest.
